# Microfiber/Nanofiber/Attapulgite Multilayer Separator with a Pore-Size Gradient for High-Performance and Safe Lithium-Ion Batteries

**DOI:** 10.3390/molecules29143277

**Published:** 2024-07-11

**Authors:** Zichen Wang, Haipeng Ren, Bo Wang, Sijing Yang, Bin Wu, Yige Zhou, Heqin Li, Zhenzhen Wei, Yan Zhao

**Affiliations:** College of Textile and Clothing Engineering, National Engineering Laboratory for Modern Silk, Soochow University, Suzhou 215123, China; zcwang2020@stu.suda.edu.cn (Z.W.); 20225215054@stu.suda.edu.cn (H.R.); 20225215069@stu.suda.edu.cn (B.W.); 20225215107@stu.suda.edu.cn (S.Y.); 20225215088@stu.suda.edu.cn (B.W.); xxxtora@163.com (Y.Z.); liheqin1212@163.com (H.L.)

**Keywords:** separators, pore-size gradient, nanofibrous membrane, nanoparticles, lithium-ion batteries

## Abstract

Lithium-ion batteries (LIBs) have an extremely diverse application nowadays as an environmentally friendly and renewable new energy storage technology. The porous structure of the separator, one essential component of LIBs, provides an ion transport channel for the migration of ions and directly affects the overall performance of the battery. In this work, we fabricated a composite separator (GOP-PH-ATP) via simply laminating an electrospun polyvinylidene fluoride-hexafluoropropylene (PVDF-HFP) nanofibrous membrane coated with attapulgite (ATP) nanoparticles onto a PP nonwoven microfibrous fabric, which exhibits a unique porous structure with a pore-size gradient along the thickness direction that ranges from tens of microns to hundreds of nanometers. As a result, besides the enhanced thermal stability given by the chosen materials, the GOP-PH-ATP separator was endowed with a superhigh porosity of ~95%, strong affinity with electrolyte, and great electrolyte uptake of ~760%, thus effectively enabling an ionic conductivity of 2.38 mS cm^−1^ and a lithium-ion transference number of 0.62. Furthermore, the cell with the GOP-PH-ATP separator shows an excellent cycling performance with a capacity retention of 91.2% after 150 cycles at 1 C, suggesting that the composite separator with a pore-size gradient structure has great potential to be applied in LIBs.

## 1. Introduction

Lithium-ion batteries (LIBs) are an environmentally friendly and sustainable new energy storage technology with a wide range of potential applications under the general trend of green energy as the development strategy [1]. An LIB is primarily composed of an anode, cathode, electrolyte, and separator, the former three of which provide and transport the lithium ions between electrodes during charge–discharge cycles [2]. The separator acts as a barrier between the electrodes and provides ion transport channels for lithium ions to migrate through, and consequently, its structure and properties directly affect the overall performance of the battery [3].

The primary material used in the current commercial separators is polyolefin; however, its low porosity (30–50% [4,5]), poor wettability, and poor thermal stability (melting at 150 °C [6]) severely limit its application in LIBs. Apart from modifying these polyolefin separators by means of coating inorganic nanoparticles (e.g., SiO_2_ [7], TiO_2_ [8,9], ZrO_2_ [10], and Al_2_O_3_ [11,12]) or polymeric materials (e.g., polyethylene (PE) [13], polyvinylidene fluoride (PVDF) [14], and poly(vinylidene fluoride-hexafluoropropylene) (PVDF-HFP) [15]), there has been significant focus on developing new separators through the utilization of novel materials, e.g., cellulose [16,17], polyimide [18,19], and/or advanced techniques like electrospinning [20,21] and vacuum filtration [22,23]. Among them, meltblown nonwoven fabric that is interwoven with numerous microfibers has come into prominence due to the advantages of easy large-scale production, the same raw materials with commercial separators, a three-dimensional pore structure with high porosity, and excellent mechanical strength. However, it only can be utilized as a separator substrate, because the pore size of the nonwoven fabric is typically at the micron level (i.e., in the range of a few microns to tens of microns) resulting from the limitation of the meltblown manufacture process, and such a large pore size cannot meet the basic requirement, often less than 1 µm, of a battery separator [24,25].

The pore size of the separator, one of the most fundamental morphological features, has a significant influence on the electrolyte wettability, the ability to transport ions, and even the cycling performance of the battery. Therefore, efforts have already been put into applying inorganic particle coating (e.g., SiO_2_ [26], Al_2_O_3_ [27], diatomite [28], and attapulgite [29,30]) to fabricate the nonwoven fabric-based composite separators with an appropriate pore size on the whole. For example, Li et al. [27] obtained an Al_2_O_3_/PET separator with high electrolyte uptake (121.5%) by simply coating a cubic Al_2_O_3_ on the surface of nonwoven fabric. Li et al. [28] prepared a polyethylene terephthalate (PET) composite separator via coating the diatomite onto the PET nonwoven fabric, which effectively reduced the pore size of the separator to 0.5 µm through the incorporation of these diatomite particles. Meanwhile, it has been demonstrated that nanoparticles also contribute to the mechanical and thermal property enhancement because of their high modulus and thermal stability [29]. For example, Yang et al. [30] immersed ethanol-treated Celgard separators in ATP-PVA solution and obtained better mechanical properties with an increase of 5.54% in tensile strength and 16.97% in elongation at break. In summary, incorporating nanoparticles into the fabric is really an effective strategy, not only in reducing the pore size and optimizing the porous structure but also in improving the electrolyte wettability and mechanical and thermal properties because of the advantages of the nanoparticles themselves [31,32].

Among the various polymeric surface modifications on nonwoven fabrics, electrospun nanofibrous membranes are also used in structural regulation due to their high porosity and uniform pore size [33,34,35]. For example, Peng et al. [36] electrospun polysulfonamide (PSA) on both sides of PET nonwoven fabric to prepare a PSA/PET/PSA separator with nanosized pores. Jeong et al. [14] formed a PVDF/PET/PVDF structure on the PET nonwoven substrate by electrospinning, with the pore size of the separator significantly reduced and a porosity of 134.5%. Zhou et al. [37] laminated a PVDF-HFP nanofibrous membrane with modified PP nonwoven fabric to prepare a separator with a porosity of 88.7% and an electrolyte uptake of 361%. Therefore, stacking the nanofibrous membrane onto the nonwoven fabric with microfibers is equivalent to constructing a nanofiber layer whose porous structure is more suitable for LIBs. Meanwhile, the pore size of the composite separator exhibits a direct change from tens of micrometers to a few micrometers or nanometers. However, constructing a more delicate gradient, e.g., gradually changing from tens of micrometers to a few micrometers to nanometers, by integrating the microfibers, nanofibers, and inorganic nanoparticles, has been rarely studied, not to mention their effects on the structure and properties of battery performance.

In this contribution, a composite separator with a pore-size gradient was proposed for LIBs with high performance and safety, which is achieved by first surface-modifying a nonwoven fabric, followed by the lamination of an electrospun membrane that has been pre-coated with a layer of nanoparticles. The effect of nanoparticle content on the porous structure, electrolyte wettability, and mechanical and thermal properties of the resultant composite separator was deeply investigated, and the influence of the pore-size gradient structure on the electrochemical and battery performance was also taken into account, hoping that the composite separator has potential to be applied in LIBs.

## 2. Results and Discussion

### 2.1. Morphological and Structural Analysis

**Figure 1:**
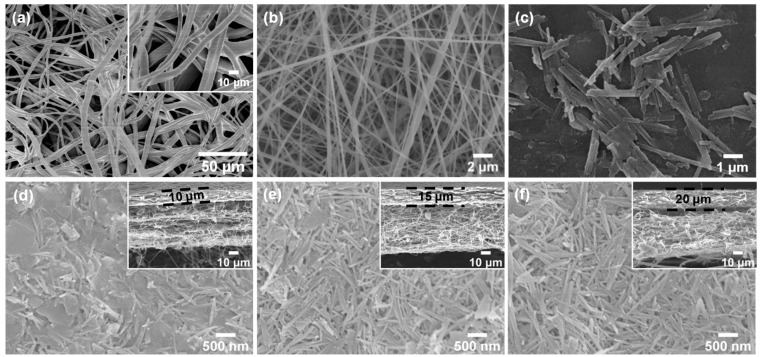
SEM images of (**a**) GOP membrane, (**b**) PVDF-HFP nanofibrous membrane, (**c**) ATP nanoparticles, (**d**) GOP-PH-9ATP, (**e**) GOP-PH-12ATP, and (**f**) GOP-PH-15ATP separators.

The monolayer morphology of each component of the GOP-PH-ATP separator can be clearly seen in Figure 1a–c. The GOP membrane (Figure 1a) had a large number of randomly oriented microscale fibers with an average diameter of ~3 μm. Compared with the SEM image of PP nonwoven fabrics in Figure S1, the porous morphology of the GOP membrane did not significantly change after the impregnation treatment with GPTE/ODA modification solution. However, there was a distinct overlay on the GOP fiber surface. It could be attributed to the formation of a reaction product by the epoxide-amino coupling reaction between GPTE and ODA catalyzed by 1-methylimidazole, which has been reported in our previous work [37]. The infrared spectra (Figure S2, Supplementary Material) of the PP and GOP membrane can verify this surface modification. New peaks can be seen at 1096 cm^−1^ and 931 cm^−1^, corresponding to the C–O–C stretching vibration and the asymmetric vibration of the epoxy group, respectively. The peaks at 2927 cm^−1^ and 2865 cm^−1^, which were assigned to the C–H stretching vibration in the alkyl group, were strengthened and the peak enhancement at 1371 cm^−1^ was correlated to the CH_3_ bending vibration. The above results further indicated that GPTE and ODA were reacted on the surface of fibers by undergoing an epoxy–amino coupling reaction, rendering the membrane with polar groups such as C–O–C and epoxy after the surface treatment. 

The PVDF-HFP nanofibrous membrane (Figure 1b) presented a porous structure that was randomly interwoven with nanofibers. In combination with its own unique chemical structure, PVDF-HFP is able to exhibit superior mechanical properties and electrochemical stability, which is why it is chosen over PVDF as the material. The ATP nanoparticles clearly displayed a unique short rod-like fiber structure, as shown in Figure 1c. When the ATP was coated on the PVDF-HFP (as shown in Figure 1d–f), the surface of the separator displayed an overlapping morphology of rod-like fibers and sheet-like structures. When a small amount of ATP was coated (GOP-PH-9ATP) on the surface, the SBR binder could produce a large number of sheet-like areas, resulting in the formation of ATP-bonded clumps and non-porous regions. As the amount of ATP coating increased, the rod-like fibers were clearly increased and irregularly arranged. Meanwhile, from the cross-section images (Figure 1d–f), it can be seen that the ATP coatings were firmly bonded to the nanofiber membrane, and the thickness of the ATP coating increased with the increase in coating amount.

**Figure 2:**
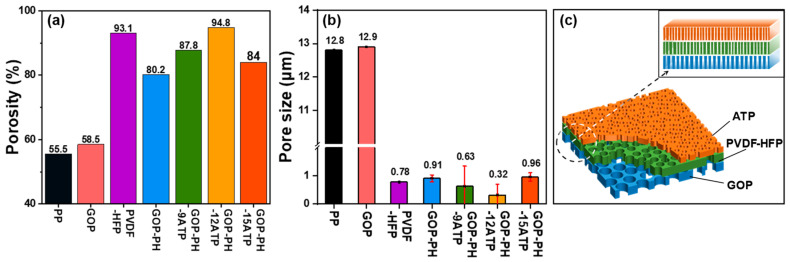
(**a**) Porosity and (**b**) average pore size of the different separators. (**c**) Schematic illustration showing the pore-size gradient of the GOP-PH-ATP separator.

To quantitatively characterize the porous structure of the separators, their porosity and average pore sizes were determined and are shown in Figure 2 and summarized in Table 1. As displayed in Figure 2a, the porosity of pure PP nonwoven fabric was 55.5% and the porosity of the GOP membrane after surface treatment was slightly increased (58.5%). This was because, during the testing process, the nonwoven substrate had reduced retention for the *n*-butanol liquid due to its large pore size. Laminated with the nanofibrous membrane, the GOP-PH separator was well modified with a porosity of 80.2%. The porosity of the GOP-PH-ATP separators all increased after ATP coating, indicating the positive role of the ATP coating layer in enhancing porosity. With the increase in ATP content, the porosity of the composite separator showed a tendency to increase first and then decrease, which was possibly related to the dispersion of ATP components in the separator. When the ATP composition was 20.7 wt% (GOP-PH-12ATP separator), the highest porosity (94.8%) was exhibited, accompanied by the ATP nanorods being bonded to the surface of the separator. When the ATP content increased, the separator showed a slightly lower porosity possibly due to the aggregation of nanoparticles. Meanwhile, it should be pointed out that because of the excessive amount and ineffective bonds between ATP and binders, as shown in Figure S3, some ATP particles of the GOP-PH-15ATP separator were shed from the separator after mechanically bending, curling, or stretching, or after being immersed in n-butanol or electrolyte, which is supposed to affect porosity and subsequent separator performance.

The average pore size and its distribution of the various separators are presented in Figure 2b and Figure S4. The average pore size of the GOP membrane (12.9 μm) remained unchanged with the PP membrane (12.8 μm), but the nonwoven is too large to be directly applicable to the separator for LIBs [24,38]. The PVDF-HFP nanofibrous membrane as the first step of pore size modification made the average pore size of the GOP-PH separator reach the nanoscale (0.91 μm). The coated layer of ATP, also as a nanoscale component, played a further role in modifying the pore size of the separator. With the increase in ATP coating amount, the average pore size of the GOP-PH-ATP separator showed a trend of increasing and then decreasing, which was similar to its effect on porosity (Figure 2). The prepared GOP-PH-12ATP separator exhibited the most superior average pore size (0.32 μm) because of the appropriate content and uniformly dispersed ATP particles on the surface, and SBR was sufficiently bonded to have a better modification effect. Therefore, the GOP-PH-ATP separator exhibited nano-micrometer pore size variation from the ATP layer to the GOP layer, forming a unique pore-size gradient structure (Figure 2c). The ATP coating produces a uniformly dispersed layer with nanoscale fine pore size. The PVDF-HFP nanofiber layer, sandwiched between the ATP and GOP, presents abundant nanoscale 3D pores with small and uniform pore size. The large pore size and high porosity of the GOP layer forms the substrate of the separator. By simply laminating the GOP layer with the ATP and PVDF-HFP layer, the separator with a pore-size gradient was thus obtained.

### 2.2. Wettability and Mechanical and Thermal Properties

**Figure 3:**
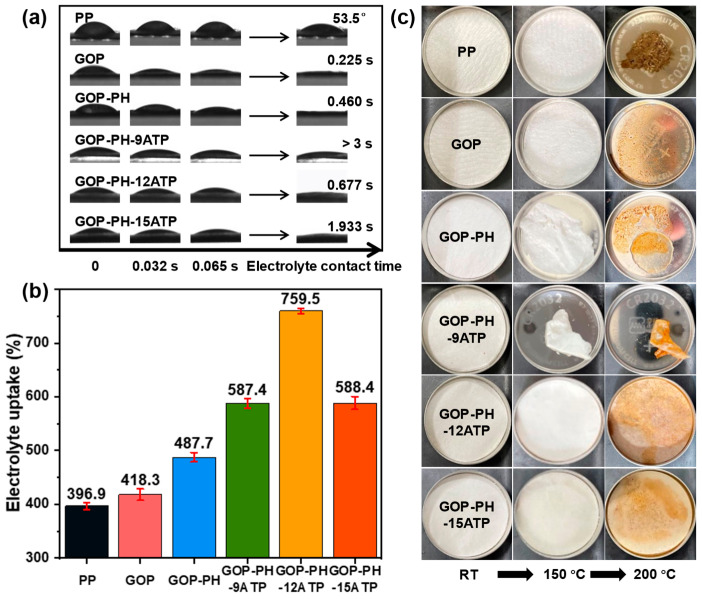
(**a**) Electrolyte contact angle, (**b**) electrolyte uptake, and (**c**) thermal stability of different separators.

Superior wettability can both speed up battery assembly and improve its cycle performance and C-rate capability. In order to examine the wettability of the separator, an electrolyte contact angle test of the separator was conducted (Figure 3a). The PP nonwoven had poor wettability with a contact angle of 53.5°. After soaking in the GPTE/ODA solution, the GOP membrane showed excellent wettability (0.225 s for complete wetting), which was attributed to the polar groups on the surface, thus improving electrolyte affinity. Complete wetting was also achieved on the ATP side of the GOP-PH-ATP separator, which could be ascribed to the polar groups of ATP and the capillary action of the nanoscale pores. Furthermore, the wetting properties of the electrolyte were not compared with those of commercial polyolefin separators due to differences in preparation methods, as well as the well-established basic parameters of commercial separators [39].

To further compare the wettability of different separators, the electrolyte uptake was tested as shown in Figure 3b and recorded in Table 1. After surface treatment, the electrolyte uptake of the GOP membrane was up to 418.3%, which was significantly higher than that of PP (396.9%), indicating that the electrolyte affinity of the separator was improved by incorporating polar groups. However, because of the oversized pore size, the electrolyte uptake was still low, resulting in an insignificant increase in the electrolyte uptake of the separator. With the addition of the PVDF-HFP nanofiber layer and the ATP nanoparticle layer, the electrolyte uptake of the GOP-PH-ATP separator (>580%) was significantly higher than those of the commercial separator and the substrate, which could be due to the electrolyte being effectively retained through the excellent specific surface area and interconnected micro-/nanopores. Similar to the effect of ATP on the porosity of the composite separator, the hydroxyl groups on the surface of the nanorods had a weak attraction to the liquid electrolyte when the ATP percentage was low. However, as the percentage was high (25.9 wt%), the ATP nanoparticles were easily shed from the surface, which made the electrolyte uptake decrease. 

The typical stress–strain curves of the composite separator were investigated and the binder’s effect was also taken into account. As shown in Figure S5, the tensile strength of the GOP membrane (2.05 MPa) was slightly lower than that of PP (2.42 MPa), while the extension at break was significantly weaker. This was due to the fact that the high curing temperature led to the weakening of the bond between the fibers during the surface treatment. Meanwhile, from the stress–strain curves of GOP-PH-12ATP, it can be seen that the maximum stress of the separator containing the SBR binder was 3.11 MPa and that of the separator without SBR was 2.26 MPa, but there is not much difference in the extension at break between them, suggesting that the tensile properties of the composite separator were improved and the positive role of the binder should not be ignored.

To evaluate the thermal properties of the separators, TG and DSC were performed (Figure S6). It is worth noting that the GOP-PH-12ATP separator displayed excellent thermal stability with stages of weight loss. The first weight loss stage at 300~360 °C was considered to be the decomposition of the GOP component, and the second weight-loss stage occurred in the range of 360 °C to 500 °C, which was interpreted as the decomposition of PVDF-HFP (Figure S6a). In the DSC curves of the GOP-PH-12ATP separator, two exothermic peaks were present at 130 °C and 155 °C, as seen in Figure S6b, corresponding to the melting of the PVDF-HFP and PP components, respectively. Figure 3c shows the thermal dimensional stability of different separators at different temperatures for 1 h. At room temperature, all separators showed regular appearance morphology. After heating to 150 °C, only the GOP-PH-12ATP and GOP-PH-15ATP separators were unchanged and without shrinkage. When the temperature was increased to 200 °C and kept for 1 h, the GOP-PH-12ATP and GOP-PH-15ATP composite separators were able to keep their dimensions intact. The shrinkage of the GOP-PH-9ATP separator was increased and the rest of the separators melted. The above results indicated that the inorganic ATP coating layer plays a significant positive role in the thermal stability of the separator [29,40]. Taking the above results into account, it can be indicated that the GOP-PH-12ATP separator was endowed with a thermal shutdown function. That is, when the temperature was in range of 130–200 °C, the pores were blocked by the melting of PVDF-HFP or PP, which could cut off the ion transport and shut down the current; at the same time, the separator maintained the dimensional integrity due to the existence of the ATP nanoparticles, and the internal short circuit could be avoided.

### 2.3. Ion Transport Capacity

**Figure 4:**
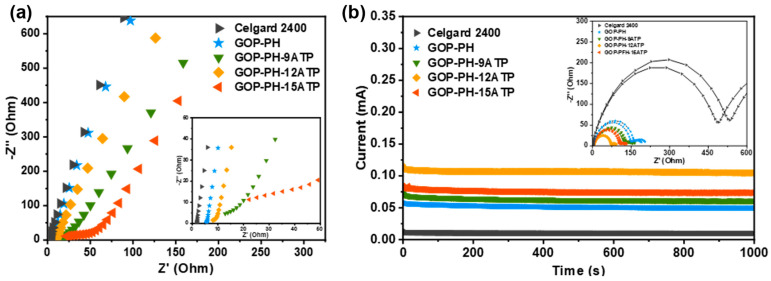
(**a**) Nyquist plots of SS/separator/SS cells using Celgard 2400, GOP-PH, and GOP-PH-ATP separators. (**b**) The potential polarization curves of Li/separator/Li cells (the insets are Nyquist plots before and after polarization).

A vital indicator characterizing the nature of electrolytes that transport ions is ionic conductivity (σ). Hence, a stainless steel (SS)/GOP-PH-ATP separator/SS cell was used to conduct electrochemical impedance spectroscopy (EIS) tests, and the Nyquist plots are displayed in Figure 4a, from which the σ values are calculated and listed in Table 1. The GOP-PH-12ATP separator showed the highest ionic conductivity (2.38 mS cm^−1^), while that of the GOP-PH-9ATP (2.03 mS cm^−1^) and GOP-PH-15ATP (1.59 mS cm^−1^) was second only to the GOP-PH-12ATP separator, and all were higher than the Celgard 2400 separator with a σ of 0.51 mS cm^−1^. In comparison to the Celgard 2400 separator, the composite separators exhibited a greater increase in ionic conductivity due to their larger thickness. However, in comparison to similar-thickness composite separators, the conductivity increase could be attributed to the improved porosity and electrolyte wettability.

**Figure 5:**
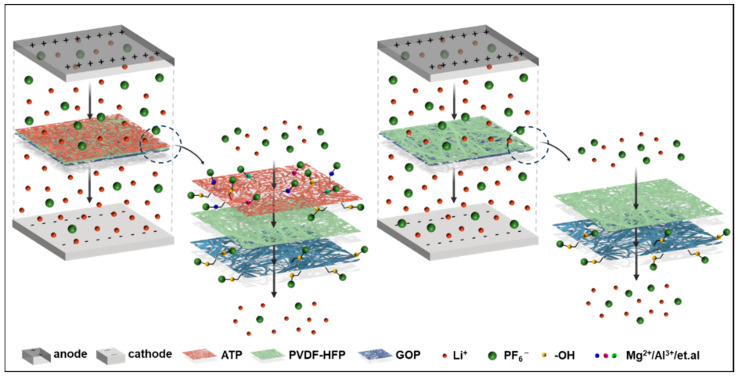
Schematic illustration for the lithium-ion transport mechanism of the GOP-PH-ATP separator.

Another important indicator of electrochemical performance is the lithium-ion transference number (*t*_Li+_). A high *t*_Li+_ value is beneficial for reducing the concentration polarization during charging and discharging and slowing down the formation and growth of lithium dendrites [41]. Figure 4b shows the DC polarization diagrams and AC impedance spectra of Li//Li symmetric cells assembled with different separators, and their *t*_Li+_ values are calculated and recorded in Table 1. The *t*_Li+_ values of the composite separators were all higher than that of Celgard 2400 (0.31) and GOP-PH separator (0.43), and the value of the GOP-PH-12ATP separator reached 0.62, which is twice that of Celgard 2400. The high lithium-ion transference number of the GOP-PH-ATP composite separator can be explained from two perspectives, and the schematic illustration for the lithium-ion transport mechanism is shown in Figure 5. From a material perspective, a large number of hydroxyl groups and metal cations in the ATP layer, like Mg^2+^ and Al^3+^, are able to capture and immobilize the anions [41,42]. The PVDF-HFP layer has a strong affinity to electrolytes and the GOP layer improves electrolyte affinity by obtaining polar groups. Capturing anions and improving the electrolyte affinity features promote lithium-ion migration. From a structural point of view, the pores with size gradient in the separator could be regarded as a large number of capillaries, and under this capillary effect, the electrolyte can be spread rapidly and largely, thus improving the lithium-ion transport capacity [43].

### 2.4. Electrochemical and Battery Performance

**Figure 6:**
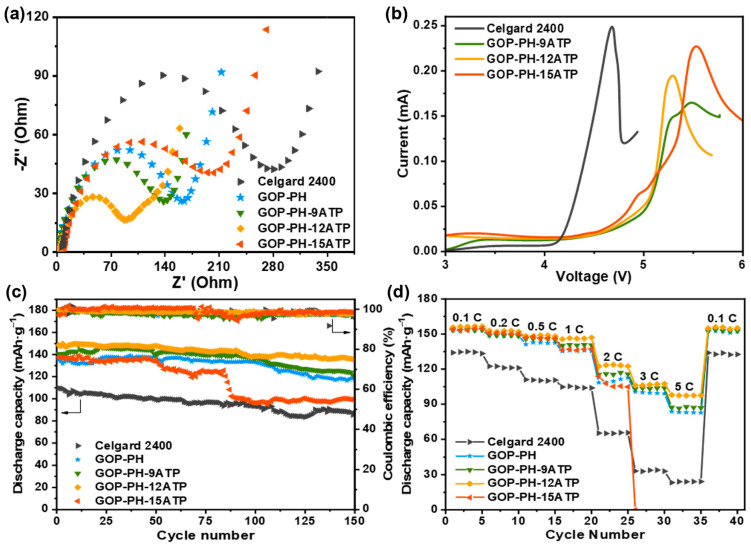
(**a**) Nyquist plots and (**b**) LSV curves of cells using different separators. (**c**) The cycle performance and (**d**) the C-rate capability of cells using different separators.

The LiFePO_4_/GOP-PH-ATP separator/Li cells for electrochemical impedance spectroscopy (EIS) testing were assembled, and the obtained interfacial resistance (*R*_in_) of different separators is shown in Figure 6a. Most of the composite separators exhibit a decrease in *R*_in_ (GOP-PH-9ATP: 156.57 Ω; GOP-PH-12ATP: 98.63 Ω) compared to GOP-PH (184.94 Ω) and Celgard 2400 (303.6 Ω). This is due to the higher electrolyte uptake and wettability of the composite separator, which results in easier electrolyte penetration to the electrode surface. There is an exception for GOP-PH-15ATP (234.73 Ω), which was due to the poor pore size distribution, low porosity, and electrolyte uptake that impedes ion transport within the separator. In addition, the presence of detached ATP also affects the interface resistance of the cell.

The electrochemical stability of the separator is an important factor in ensuring its reliability. To test the electrochemical stability of the GOP-PH-ATP separator, linear sweep voltammetry (LSV) was used (Figure 6b). It can be visualized that the electrolyte decomposition potentials of the GOP-PH-ATP separators are all higher than 5 V, which is significantly greater than that of Celgard 2400 at 3.9 V vs. Li^+^/Li. This indicates that the composite separator exhibits good electrochemical stability, probably due to the good interfacial affinity with the electrolyte, and can be applied to high-voltage LIBs [44].

Figure 6c shows the cycle performance and coulombic efficiency of the Li//LiFePO_4_ cells assembled with Celgard 2400, GOP-PH, and GOP-PH-ATP composite separators at 1 C. The initial capacities of the cells with composite separators are all higher than that of the Celgard 2400 (110 mAh g^−1^), with the GOP-PH-12ATP separator having the best initial discharge specific capacity of 149 mAh g^−1^. Furthermore, the cell with the GOP-PH-12ATP separator shows an excellent cycling performance with a capacity retention of 91.2% after 150 cycles at 1 C, and a coulombic efficiency maintained at 98%, suggesting that the composite separator effectively improves the performance and lifetime of the battery. Figure 6d shows the C-rate capability of the cells assembled with different separators under different discharge C-rate. The discharge capacities of all cells showed a decreasing trend with the increasing rate. The discharge capacity of the cell using the GOP-PH-12 ATP separator (156 mAh g^−1^ at 0.1 C, 94 mAh g^−1^ at 5 C) is significantly higher than that of Celgard 2400 (134 mAh g^−1^ at 0.1 C, 23 mAh g^−1^ at 5 C). When the rate was restored to 0.1 C, the specific capacity significantly recovered, suggesting that the cell has excellent reversibility. This can be attributed to the efficient stacking of the ATP layer nanorods in the separator, resulting in good pore size distribution and fast lithium-ion conduction. It should be noted that the cell with the GOP-PH-15ATP separator experienced a sudden drop at 85 cycles (Figure 6c) and at 26 cycles under 3 C (Figure 6d), which could possibly be attributed to the shedding of ATP particles, causing an irreversible effect on the performance stability of battery.

## 3. Experimental Section

### 3.1. Materials

Meltblown polypropylene (PP) nonwoven fabric (10 g m^−2^) was provided by Jiangsu Yingyang Nonwoven Machinery, Ltd., Changshu, China. Glycerol propoxylate triglycidyl ether (GPTE, molecular weight 434, epoxy value 0.16) was supplied by Hubei Yunmagnesium Technology Co., Ltd., Wuhan, China. Octadecylamine (ODA), 1-methylimidazole, N,N-dimethylformamide (DMF), and polyvinylidene fluoride-hexafluoropropylene (PVDF-HFP, *M*_w_ = 45,500) were supplied by Sigma-Aldrich (Shanghai, China). Attapulgite (ATP, average diameter of 30 ± 6 nm, average length of 550 ± 167 nm) was supplied by Jiangsu Gantai County Junda Attapulgite Material Co., Ltd., Huai’an, China. Carboxylic butadiene-styrene latex (SBR, solid content 49–51%) was supplied by Trinseo Polymers Co., Ltd., Zhangjiagang, China. Liquid electrolyte, 1 mol L^−1^ lithium hexafluorophosphate (LiPF_6_) in ethylene carbonate, ethyl methyl carbonate, and dimethyl carbonate (EC/EMC/DMC, 1/1/1, V/V/V), poly(vinylidene fluoride) (PVDF), carbon black (Super P-C45), LiFePO_4_, and N-methyl-2-pyrrolidone (NMP) were supplied by Ferro Corp (Mayfield Heights, OH, USA). A Celgard 2400 PP separator was used for comparison. All chemicals were used as received without further purification.

### 3.2. Preparation of GOP-PH-ATP Composite Separator

**Figure 7:**
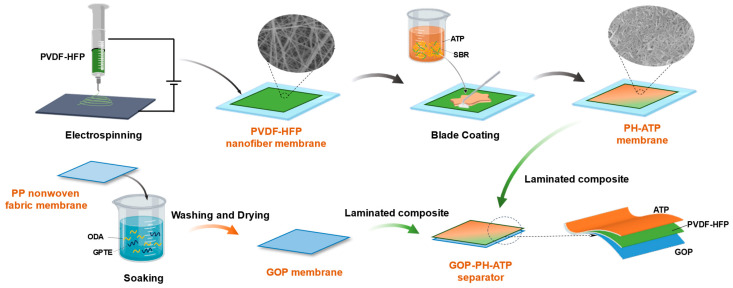
Preparation schematic diagram of GOP-PH-ATP composite separator.

The fabrication process of the composite separator is illustrated in Figure 7. Typically, the PH-ATP membrane was first prepared by electrospinning the PVDF-HFP solution, followed by blade-coating ATP nanoparticles onto the nanofibrous membrane. PVDF-HFP was dissolved in DMF with a concentration of 20 wt% and the electrospinning conditions included a voltage of 13.5 kV, a receiving distance of 15 cm, and a flow rate of 1 mL·h^−1^. A certain amount of ATP nanoparticles and binder (SBR, 0.5 wt% of ATP) were dispersed as the ATP suspension for coating. Then, the surface-modified PP nonwoven fabric (GOP membrane) was prepared as reported in our previous work [37]. Finally, the GOP-PH-ATP separator with a thickness of ~60 μm was produced by laminating the GOP membrane and the PH-ATP membrane together. Based on the amounts of ATP suspension used for coating, the resultant separators were marked as GOP-PH-9ATP, GOP-PH-12ATP, and GOP-PH-15ATP, respectively, and the specific component ratios in each separator are listed in Table 1.

### 3.3. Characterizations

The surface and cross-section morphologies of the meltblown nonwoven fabric, electrospun nanofibrous membrane, and GOP-PH-ATP composite separators were observed using a field emission scanning electron microscope (FE-SEM, Hitachi S4800, Beijing, China). The chemical compositions on the surface of the PP and GOP membranes were confirmed using a Fourier-transform infrared spectrometer (FTIR, Nicolet 5700, Waltham, MA, USA). 

The pore size of the separator was measured using the Porometer 3G through-pore size analyzer and the porosity was tested using the *n*-butanol soaking method with the following equation: $\mathrm{porosity}\;\left(\%\right)=\frac{m_{t}-m_{0}}{\rho \;V}\times 100\%$, where *m*_0_ and *m_t_* are the mass of the sample before and after it soaked in the *n*-butanol for 2 h, *ρ* is the density of *n*-butanol, and *V* is the apparent volume of the sample. The contact angles of the electrolyte, which are one indicator of wettability, were examined using a DSA100 apparatus (Krüss Company, Hamburg, Germany). The separator was immersed in the liquid electrolyte and the electrolyte uptake was characterized by measuring the weight change before and after immersion according to the following formula: $\mathrm{EU}\;\left(\%\right)=\frac{W-W_{0}}{W_{0}}\times 100\%$, where *W*_0_ and *W* are the weight of the separator before and after it absorbed the electrolyte.

The tensile strength of the separator was obtained by averaging five tests as the final result at a constant rate using an Instron Universal testing machine (Instron, 5967, Boston, MA, USA). Thermogravimetric (TG, Perkin Elmer 7, Waltham, MA, USA) analysis was conducted from 30 °C to 600 °C at a rate of 10 °C min^−1^ under nitrogen atmosphere. Differential Scanning Calorimeter (DSC, TA Instruments, New Castle, DE, USA) analysis was used to test the shutdown temperature of the separator. The thermal-dimensional stability of the separators was obtained by placing the different samples in the anode shells and testing for dimensional change at 150 °C and 200 °C.

The electrochemical performance of the separator was evaluated on an electrochemical workstation (BioLogic SP-300, Cécile Népalisse, France). The ionic conductivity of the separator was tested and compared by assembling a stainless steel (SS)/separator/SS cell using electrochemical impedance spectra (EIS) which is given by the formula: σ=d/(R×A), where *d* is the thickness of the sample, *A* is the area of the sample, and *R* represents the bulk resistance. The interfacial impedance of the separator was also researched using EIS to analyze interfacial compatibility. The lithium-ion transference number (*t*_Li+_) was determined by means of AC impedance and DC polarization techniques using Li//Li symmetric cells. The formula of *t*_Li+_ is as follows: $t_{\mathrm{Li}+}=\left(I_{s}\cdot \left(V-I_{0}R_{0}\right)\right)/\left(I_{0}\cdot \left(V-I_{s}R_{s}\right)\right)$, where *I*_0_ is the initial current value and *I_s_* is the steady-state current value, *R*_0_ is the resistance of the cell before polarization, *R_s_* is the value after polarization, and *V* is the potential of the cell. The results of the linear sweep voltammetry (LSV) test were used to characterize the electrochemical stability using an SS//Li cell at 1 mV·s^−1^ over the range of 3.0 to 6.0 V. The Li//LiFePO_4_ batteries were assembled in an Ar-filled glove box using prepared separators. The cycle performance of the cell was tested by performing 150 cycles at a rate of 1 C over a voltage range of 2.5~4.2 V. The rate discharge tests were carried out at rates of 0.1, 0.2, 0.5, 1, 2, 3, and 5 C.

## 4. Conclusions

In summary, we successfully prepared a GOP-PH-ATP composite separator by laminating the PVDF-HFP nanofibrous membrane and the ATP coating layer onto a PP nonwoven fabric. Results showed that such a multilayer stacking of organic and inorganic materials produced a separator with a highly porous structure with a pore-size gradient in the thickness direction, as well as excellent overall properties including high thermal stability, flame retardancy, and lithium-ion transport ability, with an ionic conductivity of 2.03 mS cm^−1^ and a lithium transference number of 0.62. The cell assembled with the GOP-PH-ATP separator exhibited high cycling stability with a coulombic efficiency maintained at 98% and a capacity retention of 91.2% after 150 cycles at 1 C. Therefore, the composite separator with the pore-size gradient may provide insight into the separator’s structural design and serve as an acceptable framework for the creation of LIBs that are both highly effective and safe.

## Figures and Tables

**Table 1:** Basic characteristics of separators with different ATP.

Sample	GOP-PH-9ATP	GOP-PH-12ATP	GOP-PH-15ATP
GOP (wt%)	26	23.3	21.8
PVDF-HFP (wt%)	62.5	55.8	52.3
ATP (wt%)	11.5	20.7	25.9
Thickness (μm)	55	60	62.5
Average pore size (μm)	0.63 ± 0.71	0.32 ± 0.38	0.96 ± 0.14
Porosity (%)	87.8	94.8	84
Electrolyte uptake (%)	587.4 ± 9.22	759.5 ± 5.29	588.4 ± 11.89
Ionic conductivity (mS cm^−1^)	2.03	2.38	1.59
Lithium-ion transference number	0.43	0.62	0.45

## Data Availability

Data are available from the corresponding author on request.

## Supplementary Materials

The following supporting information can be downloaded at: https://www.mdpi.com/article/10.3390/molecules29143277/s1, Figure S1. SEM image of PP nonwoven fabric; Figure S2. FTIR spectra of PP nonwoven fabric before and after wettability treatment; Figure S3. Photograph of composite separators under bending: (a) GOP-PH-12ATP, (b) GOP-PH-15ATP; Figure S4. Pore size distribution of different separators; Figure S5. Strain–stress curves of PP, GOP, GOP-PH-12ATP (without SBR), and GOP-PH-12ATP separators; Figure S6. (a) TG curves of different separators and (b) DSC curve of GOP-PH-12ATP separator.
