# Supplementary material for: Microfiber/Nanofiber/Attapulgite Multilayer Separator with a Pore-Size Gradient for High-Performance and Safe Lithium-Ion Batteries
Source: Molecules. 2024 Jul 11;29(14):3277. doi: 10.3390/molecules29143277 (PMC11279044; doi:10.3390/molecules29143277)
Supplement: Supplementary file 1 [file molecules-29-03277-s001.zip › molecules-3070788-supplementary.pdf]

Supplementary Information

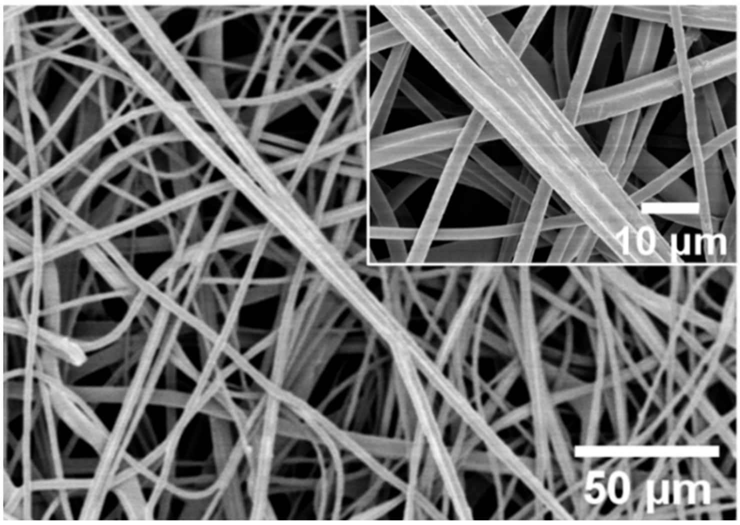

Figure S1. SEM image of PP nonwoven fabric.

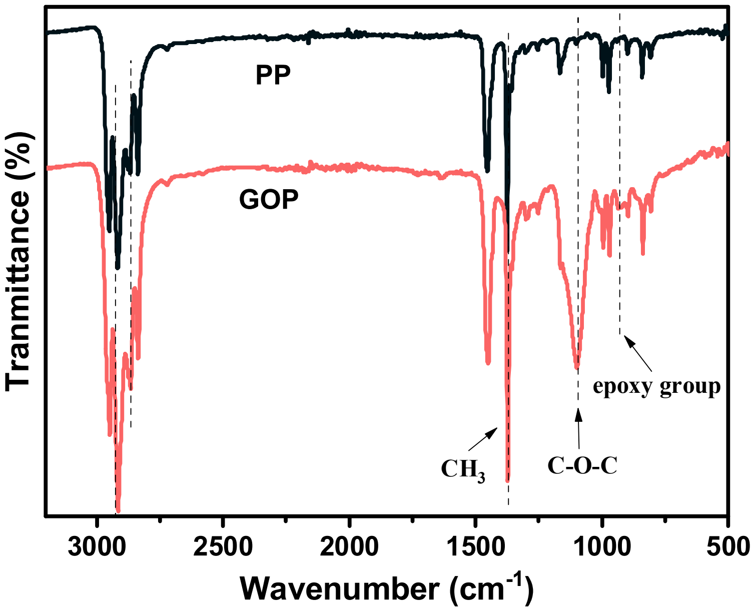

Figure S2. FTIR spectra of PP nonwoven fabric before and after wettability treatment.

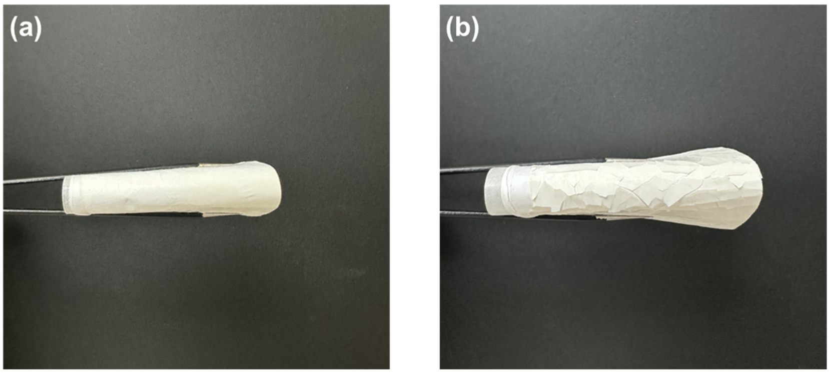

Figure S3. Photograph of composite separators under bending: (a) GOP-PH-12ATP, (b) GOP-PH-15ATP.

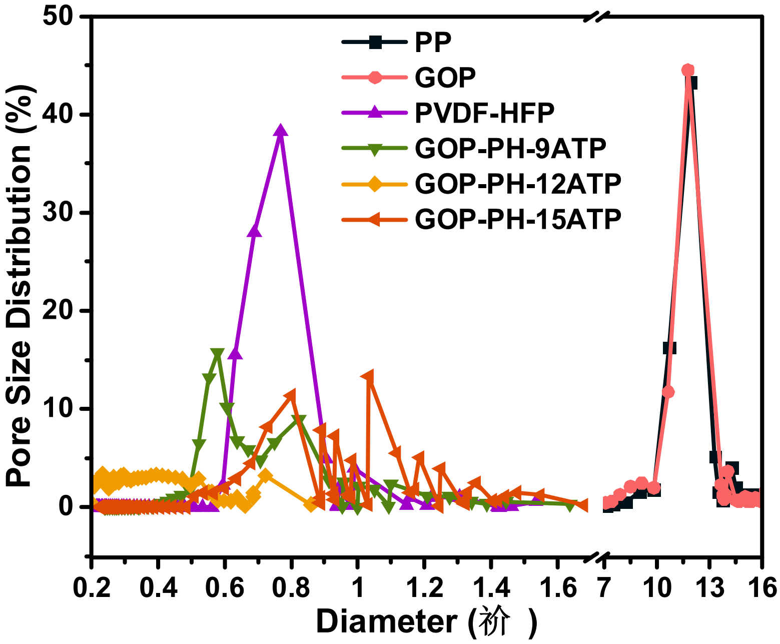

Figure S4. Pore size distribution of different separators.

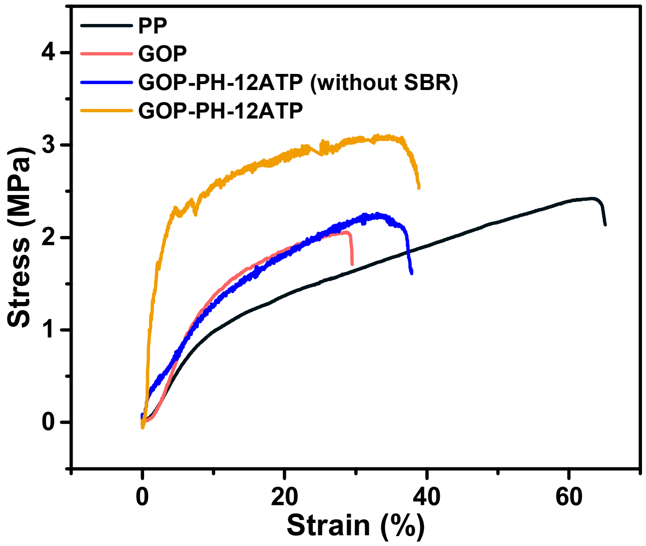

Figure S5. Strain-stress curves of PP, GOP, GOP-PH-12ATP (without SBR) and GOP-PH-12ATP separators.

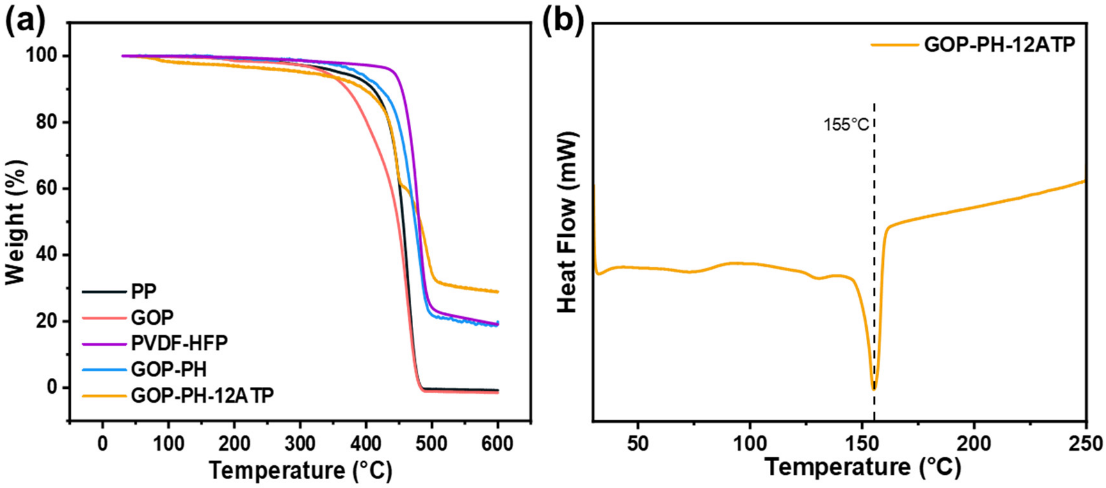

Figure S6. a) TG curves of different separators and (b) DSC curve of GOP-PH-12ATP separator.

Highlights

A composite separator (GOP-PH-ATP) with a pore-size gradient is proposed via laminating the coated electrospun nanofibrous membrane onto the modified microfiber substrate.

The GOP-PH-ATP separator exhibits greatly improved electrolyte wettability and thermal performance that were given by the chosen materials

The enhanced ion transport capacity of the composite separator is discussed from the material and structure perspectives.
